# The modifier role of *RET*-G691S polymorphism in hereditary medullary thyroid carcinoma: functional characterization and expression/penetrance studies

**DOI:** 10.1186/s13023-015-0231-z

**Published:** 2015-03-01

**Authors:** Carla Colombo, Emanuela Minna, Maria Grazia Rizzetti, Paola Romeo, Daniele Lecis, Luca Persani, Piera Mondellini, Marco A Pierotti, Angela Greco, Laura Fugazzola, Maria Grazia Borrello

**Affiliations:** Department of Clinical Sciences and Community Health, University of Milan, and Endocrine Unit, Fondazione IRCCS Ca’ Granda, Milan, Italy; Molecular Mechanisms Unit, Department of Experimental Oncology and Molecular Medicine, Fondazione IRCCS Istituto Nazionale dei Tumori, Milan, Italy; Department of Experimental Oncology and Molecular Medicine, Fondazione IRCCS Istituto Nazionale dei Tumori, Milan, Italy; Department of Clinical Sciences and Community Health, University of Milan, and Division of Endocrine and Metabolic Diseases, Ospedale San Luca, IRCCS Istituto Auxologico Italiano, Milan, Italy; Scientific Directorate, Fondazione IRCCS Istituto Nazionale dei Tumori, Milan, Italy; Department of Pathophysiology and Transplantation, Endocrine Unit, Fondazione IRCCS Ca’ Granda Ospedale Maggiore Policlinico, Milan, University of Milan, Milan, Italy

**Keywords:** *RET*, Medullary thyroid cancer, G691S, S891A, Polymorphism

## Abstract

**Background:**

Hereditary medullary thyroid carcinoma (MTC) is caused by germ-line gain of function mutations in the *RET* proto-oncogene, and a phenotypic variability among carriers of the same mutation has been reported. We recently observed this phenomenon in a large familial MTC (FMTC) family carrying the *RET*-S891A mutation. Among genetic modifiers affecting *RET*-driven MTC, a role has been hypothesized for *RET*-G691S non-synonymous polymorphism, though the issue remains controversial. Aim of this study was to define the *in vitro* contribution of *RET*-G691S to the oncogenic potential of the *RET*-S891A, previously shown to harbour low transforming activity.

**Methods:**

The *RET*-S891A and *RET*-G691S/S891A mutants were generated by site-directed mutagenesis, transiently transfected in HEK293T cells and stably expressed in NIH3T3 cells. Their oncogenic potential was defined by assessing the migration ability by wound healing assay and the anchorage-independent growth by soft agar assay in NIH3T3 cells stably expressing either the single or the double mutants. Two *RET*-S891A families were characterised for the presence of *RET*-G691S.

**Results:**

The functional studies demonstrated that *RET*-G691S/S891A double mutant displays a higher oncogenic potential than *RET*-S891A single mutant, assessed by focus formation and migration ability. Moreover, among the 25 *RET*-S891A carriers, a trend towards an earlier age of diagnosis was found in the MTC patients harboring *RET*-S891A in association with *RET*-G691S.

**Conclusions:**

We demonstrate that the *RET*-G691S non-synonymous polymorphism enhances *in vitro* the oncogenic activity of *RET*-S891A. Moreover, an effect on the phenotype was observed in the *RET*-G691S/S891A patients, thus suggesting that the analysis of this polymorphism could contribute to the decision on the more appropriate clinical and follow-up management.

**Electronic supplementary material:**

The online version of this article (doi:10.1186/s13023-015-0231-z) contains supplementary material, which is available to authorized users.

## Background

Medullary Thyroid Cancer (MTC) is a neuroendocrine neoplasia arising from thyroid parafollicular C cells. Hereditary forms account for 25% of cases and include multiple endocrine neoplasia syndromes type 2A (MEN2A), type 2B (MEN2B) and familial MTC (FMTC), caused by mutations in the rearranged during transfection (*RET*) proto-oncogene. The *RET* gene, which encodes a tyrosine kinase receptor with a crucial role in development, comprises 21 exons and generates a transcript subjected to alternative splicing leading to two main isoforms: a protein of 1114 residues displaying 51 C-terminal-specific amino acids (RET51) and a shorter protein of 1072 residues displaying nine unrelated C-terminal-specific amino acids (RET9) [1].

Beyond the known role of *RET* gain-of-function mutations in MTC, in recent years several authors investigated whether the presence of single nucleotide polymorphisms (SNPs) could be associated with susceptibility for the development or progression of MTC [2-5]. Among *RET* polymorphisms, *RET*-G691S, localized in exon 11 and reported in general population with an allele frequency of 11–33% (ARUP database “MEN2 and RET”, displaying sequence variation and clinical information [6]), is a non-synonymous variant [7-9], and could potentially affect protein function. Moreover, *RET*-G691S has been suggested to be a genetic modifier in MEN2A, related to an earlier age at presentation [7,9,10] and has been associated to the susceptibility to sporadic MTC [8,11-13], though controversial data have been reported about this issue [14].

Interestingly, *RET*-G691S has been also hypothesized to play a functional role on tumor growth and aggressiveness in pancreatic cancers and cutaneous melanoma, where it works as a genetic modifier or even as a low penetrance gene [15,16]. In particular, *RET*-G691S was reported to enhance the activation of the downstream ERK pathway compared to *RET*-wt [15], though this variant was not found to be oncogenic *per se* by focus formation assays [5,17].

Consistently with the potential modifier role of *RET*-G691S in MTC, Vandenbosch et al. observed that this polymorphism is able to enhance *RET* oncogenicity of mutations affecting RET codon 666, by increasing its penetrance in the clinical onset [18]. Accordingly, we firstly demonstrated the *in vitro* effect of the *RET*-G691S variant as enhancer of the ERK1/2 activation and of the transforming activity of the MTC-associated *RET*-K666E mutant [17].

In the present study, we investigated more deeply the potential role of this non synonymous *RET* polymorphism as a modifier of the phenotypic expression in familial MTC. To this purpose, we focused on the potential contribution of *RET*-G691S to the oncogenicity of *RET*-S891A, a *RET* mutant previously reported to be associated with low transforming activity. In particular, this gain-of-function mutation, located in the second intracellular tyrosine kinase domain of the *RET* proto-oncogene, accounts for less than 5% of all *RET* mutated patients, and can cause FMTC or the phenotype associated to MEN2A [19-23].

Since the functional characterization of *RET* mutants adds useful information to the genotyping of patients/families optimizing the diagnostic and clinical management, we both evaluated the *in vitro* biological activity of the *RET*-G691S polymorphism on the oncogenic potential of *RET*-S891A and carried out expression/penetrance studies in two unrelated *RET*-S891A FMTC families.

## Methods

### *In vitro* analyses

#### Construction of the *RET* mutants

All *RET* mutants were obtained by mutagenesis of *RET*51-wt construct (pCDNA3 vector expressing the proto-*RET* gene long isoform). *RET51*-G691S, *RET*51-C634R (containing an MEN2A causing mutation), and *RET*51-M918T (containing the main MEN2B causing mutation) used as controls are described elsewhere [17].

*RET*51-S891A and *RET*51-G691S/S891A were obtained by site-directed mutagenesis of *RET*51-WT using an *in vitro* oligonucleotide mutagenesis system (Quik-Change XL site-directed mutagenesis; Stratagene, La Jolla, CA, USA). The transition c.2071G > A leading to G691S polymorphism (SNIP rs1799939) and the transversion c.2671 T > G leading to S891A mutation were verified by DNA direct sequencing and then the mutant clones were entirely sequenced to exclude possible additional mutations. Plasmid DNA was extracted using the MAXI PREP Kit (Qiagen) as suggested by the supplier.

### Cell culture, transfections and focus formation assay

Human HEK293T cells were maintained in DMEM with 10% FCS. The recombinant plasmids were transiently transfected using Lipofectamine 2000 (Invitrogen) according to the manufacturer’s instructions.

NIH3T3 cells were cultured in DMEM supplemented with 10% serum (Colorado Company, CO). Stable transfection was performed by the CaPO4 method [24] using 100 ng plasmid DNA together with 10 μg NIH3T3-derived DNA carrier. Transfected cells were grown in DMEM with 10% serum and selected in the presence of G418 antibiotic (650 μg/ml) to obtain G418-resistant colonies indicative of transfection efficiency; transformed foci were obtained in DMEM with 5% serum in the presence of chronic stimulation with the RET ligand GDNF at a concentration of 10 ng/ml (AlomoneLabs, Jerusalem, Israel). Both G418-resistant colonies and transformed foci were fixed and counted to determine the transforming activity, calculated as foci number/colonies number ratio. NIH3T3 cell lines stably expressing *RET* mutants were derived from transformation foci picked from parallel original plates of NIH3T3 transfected cells and cultured in the presence of chronic stimulation of GDNF (10 ng/ml).

### Western blot analysis

Cells were lysed in ice-cold RIPA buffer (20 mM Tris pH 7.4, 137 mM NaCl, 10% glycerol, 0.1% SDS, 0.5% sodium deoxycholate, 1% Triton X-100, 2 mM EDTA pH 8.0) containing protease and phosphatase inhibitors.

Proteins were quantified using a modified Bradford assay (Bio-Rad). Protein samples were boiled in NuPAGE LDS sample buffer (Invitrogen), separated by NuPAGE Novex Gels with the appropriate running buffer (Invitrogen), then transferred onto nitrocellulose filters, and immunoblotted with the indicated antibodies. Anti-RET (C-20), anti-RET (H300) and anti-phospho-RET are from Santa Cruz Biotechnology (Santa Cruz, CA, USA); anti-MAP kinase (ERK1/2), anti-MAP kinase activated (pERK1/2), anti-vinculin and anti-β-tubulin are from Sigma Aldrich (Saint Louis, Missouri, USA).

Densitometric analyses were performed by the Quantity One 4.6.6 software (Bio-Rad, Hercules, CA).

### Wound healing assay

NIH3T3 cells stably expressing *RET* mutants were seeded at 30000 cells/chamber in a culture insert (Ibidi #80209) placed in 24-well plate and incubated overnight. Inserts were then removed to generate a 500 μm gap between cells. Following PBS wash and fresh medium supplement, plates were placed in the Cell-IQ SLF instrument (CM Technology Oy, Tampere, Finland) and cultured at 37°C, 5% CO_2_. Two images for well were taken each hour for 24 h using Cell-IQ Imagen software (CM Technology Oy) to monitor the gap closure. Images were then analyzed with the Scratch wound measurement tool of the Cell-IQ Analyser software (CM Technology Oy), to evaluate the % of closed area calculated with the equation: (Start wound - wound [μm^2^])/Start wound × 100. Data and graphs were analyzed using GraphPad Prism 5.02.

### Soft-agar assay

For anchorage-independent growth assay, NIH3T3 cells stably expressing *RET* mutants were suspended in DMEM containing 0.33% agar, 10% serum and GDFN 10 ng/ml (50000 cells/1.5 ml medium) and added into a layer of medium containing 0.5% agar and 10% serum in 60 mm dishes. After 3 weeks incubation, plates were analyzed for colonies number and size. Colony number was determined in plates stained with p-iodonitrotetrazolium chloride violet (Sigma Aldrich).

Agar colonies size, calculated as mean diameter, was determined by the count/size tool in Image-Pro Plus 7.0.1 software analyzing the agar colonies images the day before staining. A cutoff area of 250 pixel was manually set on NIH3T3 control cells. Only foci surpassing the cut-off area were automatically selected and scored by the software. Diameter data relative to the total foci analyzed per each field were exported and the mean diameter for each sample was calculated.

### Patients: clinical and molecular characterization

*RET*-G691S polymorphism expression/penetrance was assessed in two FMTC families carrying the germline activating mutation *RET*-S891A. Family 1 is an extremely large family with members distributed in several cities of the North of Italy, with a common ancestor [25]. Data related to some members of Family 1 have been partially reported in a previous study [23]. Family 2, from Southern Italy, is composed by 2 generations for a total of 3 subjects. Clinical and molecular features, including age at diagnosis, gender, TNM staging [26] and the disease’s outcome after a mean follow-up time of 60 months were obtained for both families. Patients were diagnosed and treated according to the American guidelines for the management of MTC [27,28]; in particular, *RET* gene carriers were screened by means of yearly basal and stimulated CT measurement and neck ultrasound. Prophylactic surgery was performed at the first evidence of a positive provocative test.

Genomic DNA was extracted from peripheral blood leucocytes of FMTC families members and the presence of *RET*-G691S polymorphism was assessed by PCR and direct sequencing as previously described [23]. All patients gave their informed consent, approved by the local Ethical Committee, to the genetic characterization and to the analysis of data.

### Statistical analysis

Statistical analysis and graphs were generated using GraphPad Prism version 5.02. Comparisons between two groups were performed by two-tailed Student’s t-test with unequal variance. A value of P < 0.05 was considered statistically significant.

## Results

### Transforming activity of *RET*-S891A and *RET*-G691S/S891A mutants

**Figure 1:**
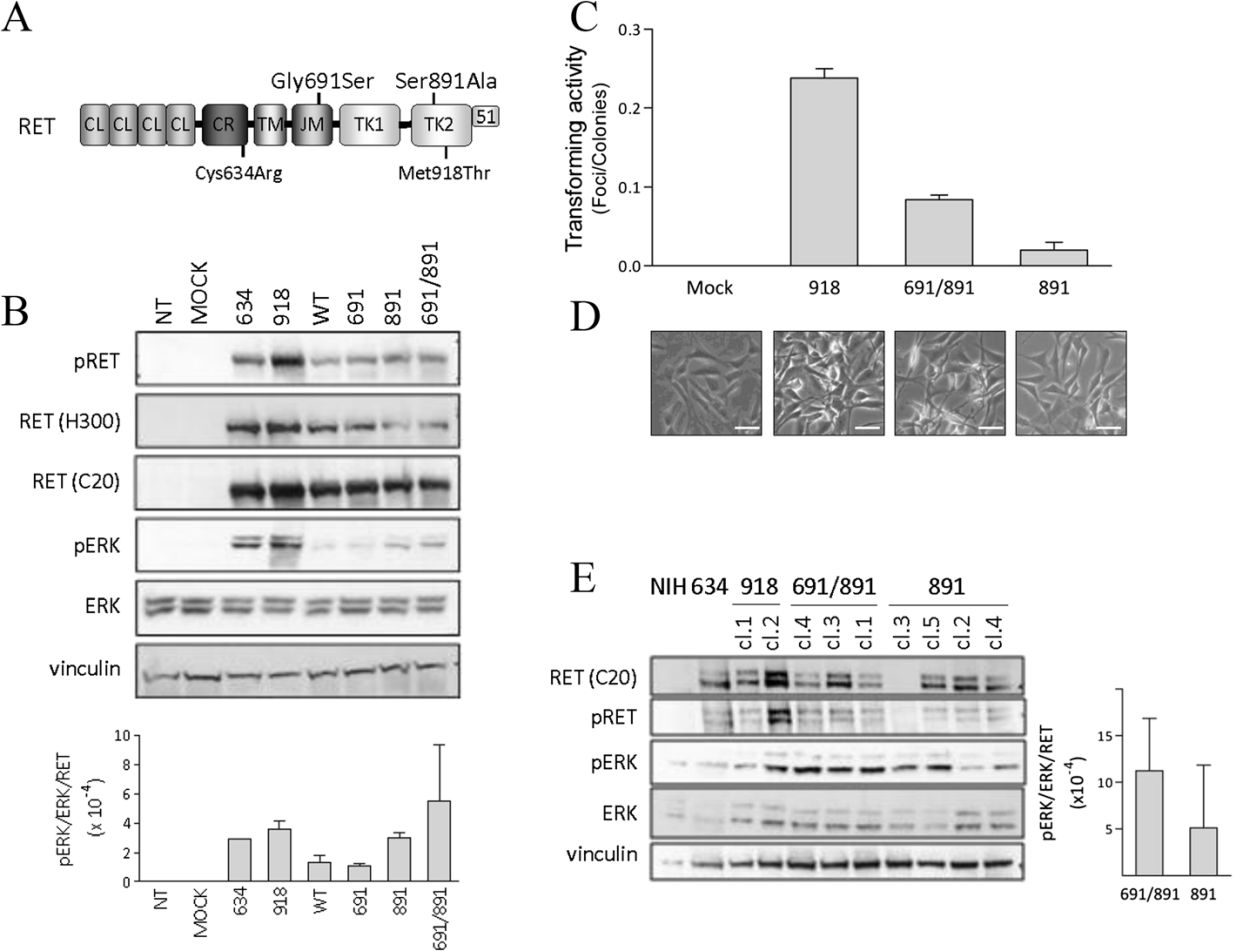
***RET***-**S891A and** ***RET*** **-G691S/S891A mutants biochemical characterization and transforming activity. A**. RET protein domains (CL, cadherin-like; CR, Cysteine-rich; TM, transmembrane; JM, juxtamembrane; TK, tyrosine kinase) and the RET51 isoform C-terminal tail are indicated. **B**. Western blot analysis of RET protein and associated ERK signaling in HEK293T cells transiently transfected. RET was detected by the antibody RET H300, specific for the RET extracellular portion, and by RET C20, specific for the intracellular portion of the RET long isoform. Vinculin is shown as protein loading control. Protein levels were quantified by densitometric analysis by Quantity One 4.6.6 software. Data are shown as mean expression value ± SD for 2 independent transfections. **C**. *RET*-S891A and *RET*-G691S/S891A mutants transforming activity by focus formation assay in NIH3T3 cells. pCDNA3 empty vector (Mock) and *RET*-M918T are included as negative and positive controls, respectively. The transforming activity, calculated as foci number/colonies number ratio, is reported as mean ± SD of triplicate counts. Data from one representative of two independent transfections are shown. **D**. Representative images of foci-derived clones. Scale bar 50um. **E**. Western blot analysis of RET protein and associated ERK signaling in NIH3T3 cells stably expressing the indicated *RET* variants: 634, *RET*-C634R; 918, *RET*-M918T; 691/891, *RET*-G691S/S891A; 891, *RET*-S891A. NIH, NIH3T3 untransfected cells. The partially and fully glycosylated forms of RET are identified in NIH3T3 cells. Vinculin is shown as protein loading control. Three *RET*-G691S/S891A clones and four *RET*-S891A clones were analysed. The ratio pERK/ERK/RET was calculated for each clone and the mean among clones ± SD was reported.

To assess the contribution of the *RET*-G691S polymorphism to the *in vitro* oncogenic potential of *RET*-S891A mutant, both localized in the intracellular domains of RET protein (Figure 1A), we generated *RET*-S891A single and *RET*-G691S/S891A double mutants by site-directed mutagenesis of recombinant plasmids carrying the *RET*-wt long isoform.

The obtained mutants, the control mutants (MEN2A-associated *RET*-C634R, MEN2B-associated *RET*-M918T, *RET*-wt and *RET*-G691S) and the empty vector were transiently transfected into HEK293T cells and the RET proteins and the associated downstream signaling on ERK1/2 were analyzed (Figure 1B). Although slightly heterogeneous, all the constructs express detectable levels of RET protein. Interestingly, the *RET*-G691S/S891A double mutant appears to activate ERK1/2 pathway more than the *RET*-S891A single mutant, as suggested by densitometric quantification (Figure 1B, lower panel).

Then, in order to analyze the biological activity of both mutants, we performed focus formation assay. The *RET*-S891A single and *RET*-G691S/S891A double mutants, the empty vector (negative control) and *RET*-M918T mutant (positive control), were stably transfected in NIH3T3 cells and the transforming activity was assessed (Figure 1C).

The *RET*-S891A single mutant shows transforming activity lower than the well known *RET*-M918T mutant, confirming previous report that indicates *RET*-S891A mutation associated to low transforming activity [21]. Interestingly, *RET*-G691S/S891A double mutant displays a threefold higher transforming activity than *RET*-S891A single mutant (0.09 *vs.* 0.03 respectively), suggesting for this polymorphism a role of transforming potential enhancer. This is also suggested by the cells morphology (Figure 1D). Indeed, whereas *RET*-S891A-derived cells display an apparently more flat morphology, *RET*-G691S/S891A-derived cells show a spindle shape morphology with contact inhibition loss, more similar to *RET*-M918T-derived control cells.

Western blot analyses performed on stable clones derived from transfection foci show that cells expressing the *RET*-G691S/S891A double mutant display on average higher levels of ERK1/2 phosphorylation, indicative of ERK1/2 pathway activation, than *RET*-S891A single mutant (Figure 1E), similarly to what shown in transient transfections (Figure 1B, lower panel).

### Cells expressing either *RET*-S891A or *RET*-G691S/S891A display different migratory and anchorage-independent growth abilities

**Figure 2:**
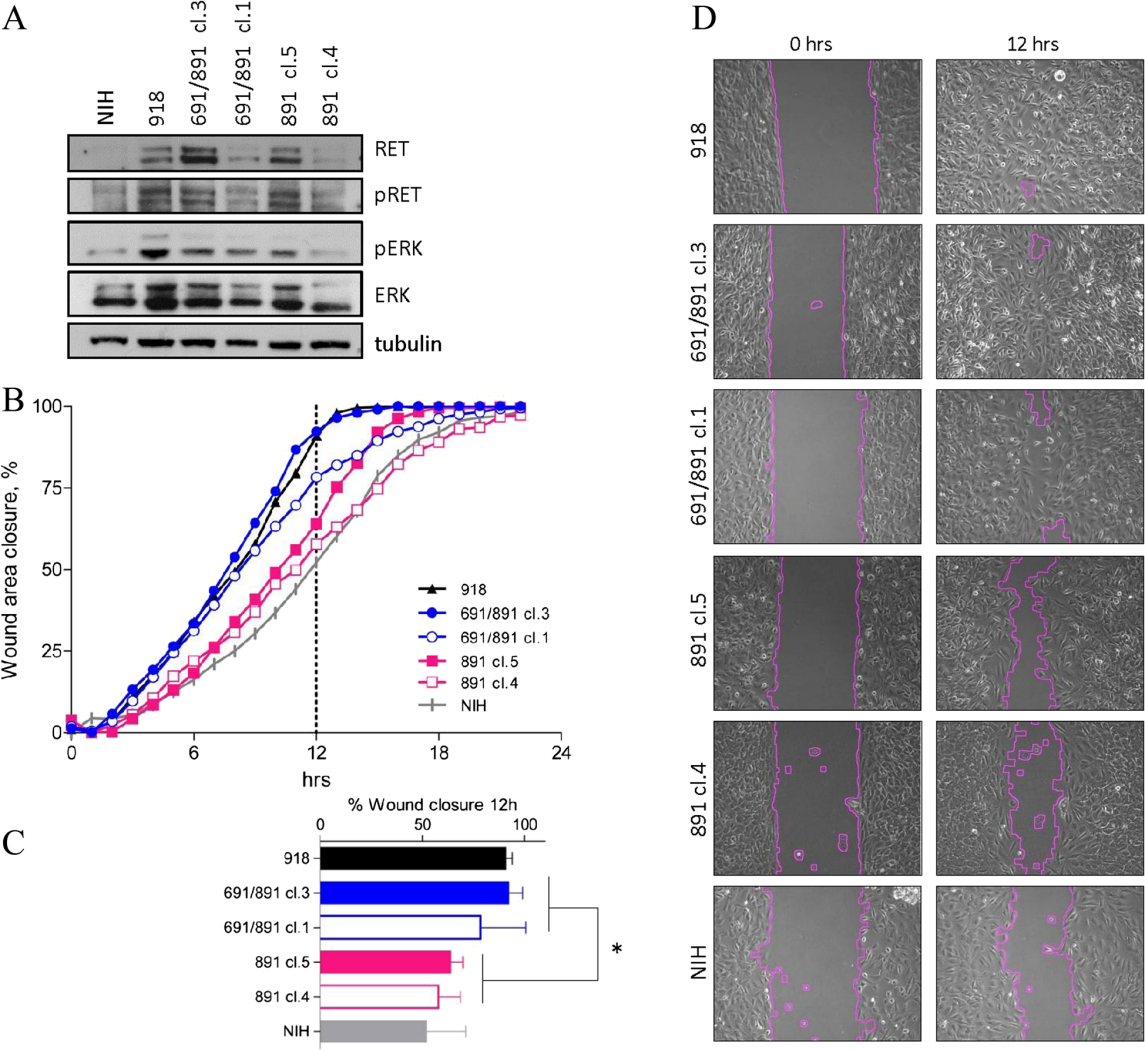
***RET***-**S891A and** ***RET*** **-G691S/S891A stable mutants migration ability by wound healing assay. A**. Western blot analysis of RET protein and associated ERK signaling in NIH3T3 cells stably expressing either *RET*-S891A or *RET*-G691S/S891A. RET was detected by RET C20 primary antibody. Tubulin is shown as protein loading control. Two clones expressing different levels of RET were analyzed for both variants. NIH3T3 cells and *RET*-M918T-expressing cells are included as negative and positive controls, respectively. **B**. Wound-healing assay in *RET* stable mutants. The wound closure percentage was quantified each hour for 24 h post-wound. Graphs are mean of two independent experiments performed in duplicate. **C**. Wound closure percentage at 12 h post-wound. Data are shown as mean ± SD of two independent experiments performed in duplicate. Statistical significance determined by Two-tailed Unpaired t test. *p < 0.05. **D**. Representative images at 0 h and 12 h post-wound (x10).

To better characterize the tumorigenic properties of *RET*-S891A single and *RET*-G691S/S891A double mutants, we performed the wound healing assay (Figure 2). The wound closure capacity, indicative of migration ability, was assessed for both mutants in two representative clones expressing different levels of RET protein (Figure 2A). Data indicate that *RET*-S891A single mutants display a statistically significant reduced capacity to close the wound compared with the *RET*-G691S/S891A double mutants. Of note, the presence of the 691S variant enhances the wound closure ability in clones expressing both high levels of RET (691/891 clone 3 vs. 891 clone 5) and low levels of RET (691/891 clone 1 vs. 891 clone 4).

**Figure 3:**
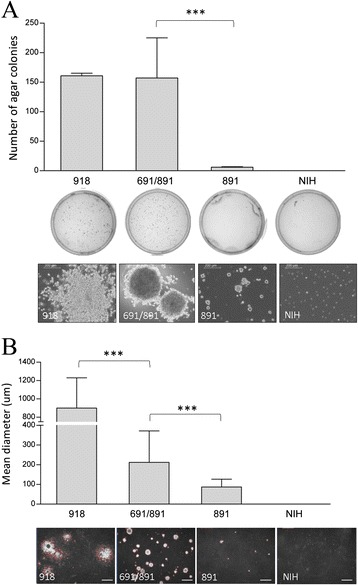
***RET*** **-S891A and** ***RET*** **-G691S/S891A stable mutants anchorage-independent growth ability by soft-** **agar assay. A**. Agar colonies in NIH3T3 cells stably expressing either *RET*-S891A or *RET*-G691S/S891A. NIH3T3 cells and *RET*-M918T-expressing cells are included as negative and positive controls. The mean ± SD of duplicate counts is reported. For *RET*-S891A and *RET*-G691S/S891A mutants two independent clones were analyzed and the mean between clones is reported. Data from one representative of two independent soft-agar assays are shown. Below representative images of the plates scored for agar colonies number and the corresponding pictures of agar colonies the day before fixing and staining (scale bar 200 um). **B**. Agar colonies mean diameter determined by Image-Pro Plus 7.0.1 software. Data are presented as mean diameter ± SD for each sample. For *RET*-S891A and *RET*-G691S/S891A two independent clones were analyzed and the mean between clones is reported. ***P < 0.0001 statistical significance determined via Student’s t-test. Below representative images of agar colonies used for diameter measurement (LEICA inverted microscope, scale bar 1000 μm).

Moreover, the anchorage-independent growth ability was assessed by soft agar assay in cells expressing the single and double mutants (Figure 3). On average *RET*-G691S/S891A double mutants give rise to a statistically significant higher number of agar colonies than *RET*-S891A single mutants (Figure 3A). In addition, the size of agar colonies originated by *RET*-G691S/S891A double mutants results statistically significant higher than those generated by *RET*-S891A single mutants (Figure 3B). To be noted, *RET*-M918T mutant generates agar colonies bigger than *RET*-G691S/S891A double mutants in agreement with its reported high oncogenic potential. These results indicate that in *RET* mutants the presence of the G691S polymorphism enhances the oncogenic properties, such as the migration and the agar colony formation ability.

### Allele frequency of *RET*-G691S polymorphism in FMTC cases harboring *RET*-S891A mutation and correlation between genetic and clinical features

The *RET*-S891A germline mutation was present in 39/69 members of the two FMTC families analyzed, whereas the *RET*-G691S polymorphism was documented in 16/69 cases (23.2%). Among the 39 *RET*-S891A carriers, 14 also harbored the *RET*-G691S polymorphism either in heterozygosis (n = 13) or in homozygosis (n = 1), showing an overall allele frequency of 19% (15 mutated alleles out of 78 alleles). Thus, in the present series, the prevalence of the co-segregation *RET*891/*RET*691 was similar to that reported in general population by the publicly available ARUP database [6]. Interestingly, *RET*-G691S appears to display a *trans* presentation with *RET*-S891A in Family 1, whereas a *cis* presentation is observed in Family 2 (Table 1). In 25/39 *RET*-S891A carriers, the clinical records were available, and they were thus classified according to the histologic diagnosis: 13 MTC, 6 CCH and 6 non C-cells disease (Table 1).

**Table 1:** **Features and 691 polymorphic status of patients harboring the** ***RET***-**S891A germline mutation**

	**Patient**	**Gender**	**Age at Dx (years)**	**RET 691**	**691 variant**	**Diagnosis**	**TNM**	**Stage**
**Family 1**	6 TL	M	15	G/G	wt	Normal	-	-
	7 RA	M	18	G/G	wt	Normal	-	-
	1 BS	M	5	G/S	polymorphic	Normal	-	-
	2 RF	M	7	G/S	polymorphic	Normal	-	-
	4 RM	M	9	G/S	polymorphic	Normal	-	-
	10 RE	F	27	G/S	polymorphic	Normal	-	-
	3 TM	M	8	G/G	wt	CCH	-	-
	8 MM	M	24	G/G	wt	CCH	-	-
	21 RP	M	56	G/G	wt	CCH	-	-
	5 BM	M	10	G/S	polymorphic	CCH	-	-
	9 RL	M	24	G/S	polymorphic	CCH	-	-
	11 RI	F	27	G/S	polymorphic	CCH	-	-
	16 FE	F	42	G/G	wt	MTC	pT1N0M0	I
	17 BA	F	43	G/G	wt	MTC	pT1N0M0	I
	18 FA	M	43	G/G	wt	MTC	pT1N0M0	I
	19 BC	F	46	G/G	wt	MTC	pT3N1bM0	IVA
	20 MC	F	54	G/G	wt	MTC	pT2N1aM0	III
	22 RU	M	62	G/G	wt	MTC	pT1NXM0	I
	25 TG	F	76	G/G	wt	MTC	pT1N1aM0	III
	12 RS	F	29	G/S	polymorphic	MTC	pT1NXM0	I
	23 RL	F	71	G/S	polymorphic	MTC	pT2NXM0	II
	13 RF	M	30	G/S	polymorphic	MTC	pT1N1aM0	I
**Family 2**	14 VV	F	35	G/S	polymorphic	MTC	pT1NXM0	I
	15VN*	M	38	G/S	polymorphic	MTC	pT1NXM0	I
	24 SV*	F	74	S/S	polymorphic^†^	MTC	pT2N1aM0	III

Age at Dx, age at diagnosis; G/G, Glycine/Glycine; G/S, Glycine/Serine; S/S, Serine/Serine; wt, wild type; Normal, absence of C-cells disease; CCH, C cells hyperplasia; MTC, medullary thyroid cancer;

*elevated urinary normetanephrine levels.

^†^RET-G691S polymorphism in homozygosis.

The Table includes the 25 patients for whom clinical data are available.

Interestingly, even though not statistically significant, a trend towards an earlier age at diagnosis was found in MTC patients harboring the *RET*-G691S polymorphism either considering MTC patients of Family 1 (median age 30 vs. 46 years; Additional file 1: Figure S1A), or the two families all together (median age 36.5 vs. 46 years; Additional file 1: Figure S1B) thus confirming *in vivo* the role of this polymorphism in the enhancement of the oncogenic activity of *RET*-S891A. Consistently, 2/3 members of Family 2 carrying *RET*-G691S/S891A had elevated urinary normetanephrine levels (#VN 445.3 mcg/24 h, nv <390 μg/24 h and #SV 578 mcg/24 h, nv <354 μg/24 h), though no lesion suspicious for pheochromocytoma (PHEO) have been yet identified using specific imaging screening (CT scan, FDG PET and MIBG scintiscan). Finally, no significant differences were noted in the outcome of patients with or without *RET*-G691S, likely as a consequence of the bias introduced by prophylactic thyroidectomy in gene carriers.

## Discussion

In this study we demonstrated for the first time that the functional polymorphic variant *RET*-G691S, not oncogenic *per se*, enhances the *in vitro* oncogenic potential of the *RET*-S891A, a mutant causing hereditary MTC. Furthermore, we showed in two FMTC families that carriers of *RET*-S891A mutation associated with the *RET*-G691S polymorphic variant display a trend towards an earlier age at diagnosis of MTC and the adrenal medulla involvement.

*RET* mutations in hereditary MTC are a paradigmatic example of clinical decision based on molecular diagnosis. Nevertheless, even within families and patients with the same *RET* mutation, the age of disease onset and the phenotypes are unpredictable, suggesting the existence of modifier loci influencing the expression and severity of the disease whose identification could drive the diagnostic and therapeutic management. Accordingly, though *RET*-S891A mutation has been associated to isolated FMTC for several years after its first identification in 1997 [29], a MEN2 clinical spectrum has been more recently reported in around 4% of cases [19]. Interestingly, a recent case report paper identify bilateral pheochromocytoma as the first manifestation of MEN2A disease in a patient of a family carrying the *RET*-S891A mutation [30].

Among the potential genetic modifiers, *RET* gene polymorphisms have been associated with susceptibility and/or disease progression [2-5], and the attention has been mainly focused on the non-synonymous variant *RET*-G691S. Although debated, *RET*-G691S has been suggested to have a role in MTC susceptibility and in the modulation of the age of onset [7-9,13,31]. In this context we recently showed that *RET*-G691S, not oncogenic *per se*, enhances *in vitro* the oncogenic potential of the rare germline mutation *RET*-K666E [5,17], accordingly with the observation of Vandenbosch et al. that this polymorphism is able to increase the penetrance in the clinical onset of mutations affecting *RET* 666 codon [18].

In the present work, to get more insights and to confirm the possible role of *RET*-G691S in the modulation of the oncogenic capacity and phenotype expression of *RET* mutants, we focused on the *RET*-S891A. This intermediated risk mutation displays *in vitro* low transforming activity [21] that may be enhanced by GDNF treatment (data not shown), as previously reported for other *RET* mutations localized in the tyrosine kinase domain, such as *RET*-M918T causally related to MEN2B [32]. In agreement with our initial hypothesis, we showed in this study that also for *RET*-S891A mutation the association with *RET*-G691S polymorphism correlates *in vitro* with enhanced tumorigenic properties such as transforming activity and migratory and clonogenic ability. These findings, indicating a possible role of genetic modifier for this polymorphism, are concordant with the expression/penetrance pattern of the two analyzed *RET*-S891A FMTC families, that include members harboring the *RET*-S891A mutation associated or not with the *RET*-G691S polymorphism. Though not statistically significant, an earlier age at diagnosis of MTC was observed in *RET*-G691S/S891A carriers compared with *RET*-S891A carriers, suggesting a possible role in the modulation of the age at disease onset.

Our *in vitro* studies, mimicking the *cis* presentation of *RET*-S891A mutation and *RET*-G691S polymorphism observed in the Family 2, demonstrate the oncogenic enhancer role of the polymorphism in the case of *cis* presentation. Moreover, the clinical data from both families suggest a trend towards an earlier age at diagnosis of MTC in the *RET*-G691S/S891A carriers irrespectively of the *cis* or *trans* presentation of the *RET*-S891A mutation and the *RET*-G691S polymorphism (Additional file 1: Figure S1B). Therefore, it is conceivable to hypothesize that the *RET*-G691S polymorphism may act as genetic modifier also in the case of *trans* presentation with *RET*-S891A mutation. However, further studies are necessary to demonstrate that *RET*-*G*691S polymorphism may enhance the oncogenic activity of *RET*-S891A, or of other *RET* mutants, also in trans.

Moreover, clinical data from Family 2 indicate that two out of three *RET*-G691/S891A patients had elevated normetanephrine levels, suggesting that the full expression of MEN2A phenotype could be related to the association with this non synonymous polymporphism, especially in the case of *cis* presentation with *RET*-S891A mutation.

## Conclusion

In conclusion, *RET*-G691S polymorphism has been shown *in vitro* to enhance the oncogenic potential of the *RET*-S891A mutation and *in vivo* to modify the clinical expression of the disease.

The genetic analysis of *RET*-G691S in other series with *RET*-S891A or other *RET* mutations is desirable to confirm the role of this polymorphism in the modulation of the phenotype, particularly in intermediate risk forms. If confirmed, present data suggest that the evaluation of *RET*-G691S in *RET* carriers should be always recommended in order to select the more appropriate clinical and follow-up management.

## Additional file

Additional file 1: Figure S1. Age distribution of *RET*-S891A MTC patients harboring or not the polymorphism. The age at the diagnosis (age at DX) and the median are reported for MTC patient from Family 1 **(A)** and for MTC patient from both families analyzed together **(B)**. The comparison between groups, performed by Wilcoxon rank sum test, results not statistically significant. The two *RET*-G691S/S891A MTC patients of family 2 displaying elevated urinary normetanephrine levels are marked (red asterisks).

## Abbreviations

MTC: Medullary Thyroid Cancer
RET: REarranged during Transfection
MEN: Multiple Endocrine Neoplasia
FMTC: Familial Medullary Thyroid Cancer
ERK: Extracellular signal-Regulated Kinase
DMEM: Dulbecco’s Modified Eagle Medium
G418: Geneticin selective antibiotic
GDNF: Glial cell line-Derived Neurotrophic Factor
TNM: Tumor-Nodes-Metastasis
PHEO: Pheochromocytoma
