# Supplementary figures and images for: The modifier role of RET-G691S polymorphism in hereditary medullary thyroid carcinoma: functional characterization and expression/penetrance studies
Source: Orphanet J Rare Dis. 2015 Mar 1;10:25. doi: 10.1186/s13023-015-0231-z (PMC4373282; doi:10.1186/s13023-015-0231-z)

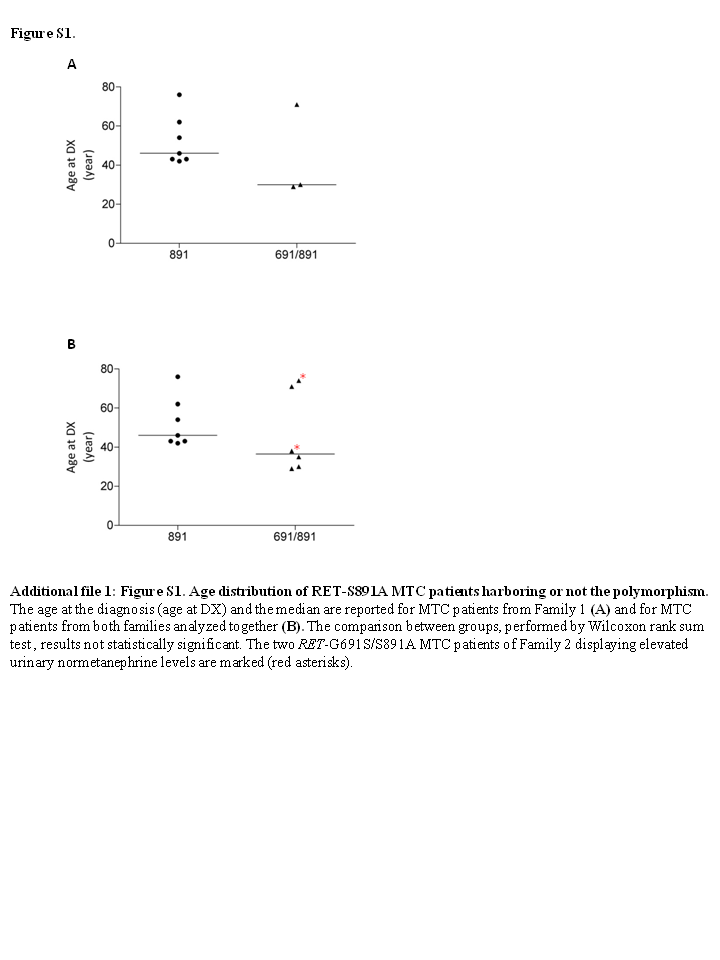

Supplement: Additional file 1: Figure S1. — Age distribution of RET-S891A MTC patients harboring or not the polymorphism. The age at the diagnosis (age at DX) and the median are reported for MTC patient from Family 1 (A) and for MTC patient from both families analyzed together (B). The comparison between groups, performed by Wilcoxon rank sum test, results not statistically significant. The two RET-G691S/S891A MTC patients of family 2 displaying elevated urinary normetanephrine levels are marked (red asterisks). [file 13023_2015_231_MOESM1_ESM.tif]
